# Presepsin in Human Milk Is Delivery Mode and Gender Dependent

**DOI:** 10.3390/nu16152554

**Published:** 2024-08-03

**Authors:** Ebe D’Adamo, Chiara Peila, Mariachiara Strozzi, Roberta Barolo, Antonio Maconi, Arianna Nanni, Valentina Botondi, Alessandra Coscia, Enrico Bertino, Francesca Gazzolo, Ali Saber Abdelhameed, Mariangela Conte, Simonetta Picone, Marianna D’Andrea, Mauro Lizzi, Maria Teresa Quarta, Diego Gazzolo

**Affiliations:** 1 Neonatal Intensive Care Unit, “G. D’Annunzio” University, 66100 Chieti, Italy; ebe.dadamo@yahoo.com (E.D.); arianna.nanni.98@gmail.com (A.N.); valentina.botondi91@gmail.com (V.B.); conte.mariangela@virgilio.it (M.C.); mary.dandrea1988@libero.it (M.D.); mauro.lizzi.med@gmail.com (M.L.);; 2 Neonatology Unit, Department of Public Health and Pediatrics, University of Turin, 10124 Torino, Italy; chiara.peila@unito.it (C.P.); roberta.barolo@unito.it (R.B.); alessandra.coscia@unito.it (A.C.); enrico.bertino@unito.it (E.B.); 3 Department of Pediatrics and Neonatology, Cardinal Massaia Hospital, 14100 Asti, Italy; chiara.strozzi@libero.it; 4 Department of Maternal, Fetal and Neonatal Medicine, ASO SS Antonio, Biagio and C. Arrigo, 15121 Alessandria, Italy; amaconi@ospedale.al.it; 5 Department of Medical and Surgical Sciences, Magna Graecia University, 88100 Catanzaro, Italy; francesca.gazzolo@libero.it; 6 Department of Pharmaceutical Chemistry, College of Pharmacy, King Saud University, Riyadh 11451, Saudi Arabia; asaber@ksu.edu.sa; 7 Neonatal Intensive Care Unit, Policlinico Casilino, 00169 Rome, Italy; simpico@libero.it

**Keywords:** presepsin, breastmilk, newborn, nutrition, preterm, sCD14

## Abstract

Breast milk (BM) is a unique food due to its nutritional composition and anti-inflammatory characteristics. Evidence has emerged on the role of Presepsin (PSEP) as a reliable marker of early sepsis diagnosis. In the present study, we aimed to investigate the measurability of PSEP in BM according to different maturation stages (colostrum, C; transition, Tr; and mature milks, Mt) and corrected for delivery mode and gender. We conducted a multicenter prospective case–control study in women who had delivered 22 term (T) and 22 preterm (PT) infants. A total of 44 human milk samples were collected and stored at −80 °C. BM PSEP (pg/mL) levels were measured by using a rapid chemiluminescent enzyme immunoassay. PSEP was detected in all samples analyzed. Higher (*p* < 0.05) BM PSEP concentrations were observed in the PT compared to the T infants. According to the grade of maturation, higher (*p* < 0.05) levels of PSEP in C compared to Tr and Mt milks were observed in the whole study population. The BM subtypes’ degrees of maturation were delivery mode and gender dependent. We found that PSEP at high concentrations supports its antimicrobial action both in PT and T infants. These results open the door to further studies investigating the role of PSEP.

## 1. Introduction

There is general consensus that breast milk (BM), due to its unique properties, constitutes the ideal product for the feeding of newborns [1,2,3]. The data in the literature support the notion that exclusive breastfeeding for the first 4–6 months of life is advised to ensure healthy growth and primary prevention against potential infectious pathogens at a stage when the neonatal immune system is completing its development [4,5]. BM is a specific–dynamic biofluid since its maturation is time-dependent and varies according to maternal diet and diseases [6]. Health benefits associated with breastfeeding lie in the combined action of the nutritional and bioactive components of BM [7,8,9,10,11,12,13,14], among which the glycoprotein CD14 aids neonatal gut function and development, modulates immune function, and regulates inflammation [15,16]. Membrane CD14 (mCD14) is a multifunctional glycoprotein expressed on the surface of various cells including monocytes, macrophages and neutrophils. Indeed, CD14 is a recognition receptor for Gram-negative and Gram-positive bacteria and lipopolysaccharides [17]. Notably, the soluble cluster of differentiation CD14 sub-type (sCD14), also called Presepsin (PSEP), has recently been put forward as a promising biomarker of sepsis [18]. Increased PSEP levels have been found in response to existing infections in adults, in children, and in infants [19,20]. In the perinatal period, the properties of PSEP, as reliable early diagnostic indicators of sepsis, can regard: (i) the fact that acute and chronic hypoxia insults do not constitute confounding factors as to its reliability in sepsis diagnosis [21]; (ii) its measurability in different biological fluids including urine and saliva [22], under several pathological conditions such as early neonatal sepsis, perinatal asphyxia, and fetal chronic hypoxia [23]; and (iii) its thermostability at room temperature and after thawing [24]. While PSEP has been established as a marker for sepsis in various biological fluids, its role and variability in breast milk across different stages of lactation and its impact on neonatal health remain unexplored.

Therefore, in the present study we aimed to investigate whether, in a cohort of preterm and term infants fed with BM, PSEP (i) was measurable in BM, (ii) varied according to different maturation stages (colostrum, transition, and mature milks), and (iii) was gestational age and gender dependent.

## 2. Materials and Methods

### 2.1. Study Population

**Figure 1:**
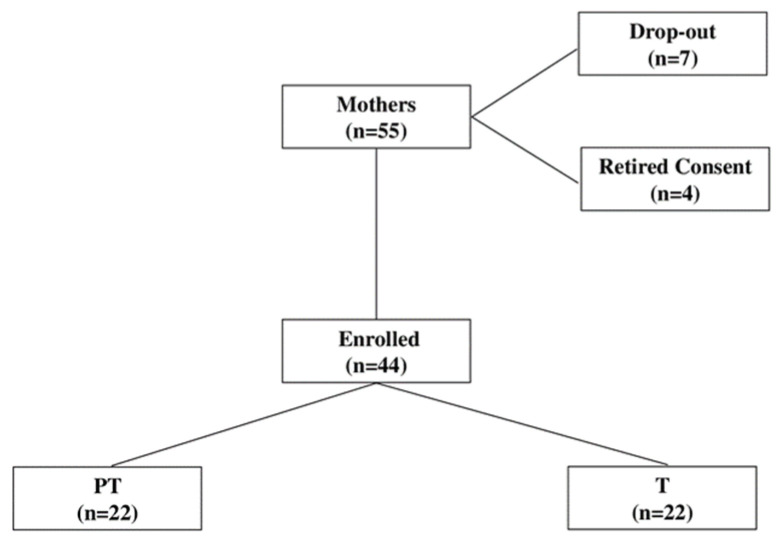
Flow chart describing patients’ recruitment. Abbreviations: PT, preterm; T, Term.

Between March 2022 and December 2023, we conducted a multicenter prospective case–control study in 55 women who delivered term (T; n = 22) and preterm (PT; n = 22) infants at our third level referral centers for neonatal intensive care (NICU). The local ethics committees (Presap.ASO.Neonat.19.02/23.05.19) approved the study and informed and signed consent was obtained from all parents of the subjects before inclusion in the study. For sample size calculation, we used changes in PSEP as the main parameter [25]. As no basic data are available for PSEP levels in BM, we assumed an increase of 0.5 standard deviation (SD) in PSEP to be clinically significant. Considering an α = 0.05 and using a two-sided test, we estimated a power of 0.95, recruiting 18 PT and T infants fed with BM. To allow for dropouts and withdrawn consent, we added 4 cases. Therefore, the study population consisted of n = 22 PT and T mothers from whom milk (colostrum, C; transition, Tr; mature, Mt) samples, defined according to Playford et al. [25], were collected from birth up to 30 days of age (Figure 1).

Gestational age (GA) was determined using clinical data and a first trimester ultrasound scan. Appropriate growth was defined by the presence of ultrasonographic signs in accordance with current guidelines, and by a post-natal confirmation of a birthweight (BW) ranging between the 10th and 90th percentiles according to our population standards, after adjusting for the mother’s height, weight, parity, and the sex of the newborn [26].

At birth, healthy newborns were defined in agreement with the criteria of the American Academy of Pediatrics [27]: no maternal illness, no signs of fetal distress, a pH greater than 7.2 in cord or venous blood, and Apgar scores greater than 7 at 1 and 5 min.

Exclusion criteria were infants with any malformations, chromosomal abnormalities, cardiac or hemolytic disease, chorioamnionitis, maternal systemic infection/inflammation, maternal fever, premature rupture of membranes, early and late onset sepsis, mastitis, and other diseases of the breast.

### 2.2. Perinatal Outcomes

The following main perinatal and neonatal outcomes were recorded in the studied groups: maternal age; gestational diabetes mellitus (GDM); pregnancy induced hypertension (PIH) and intrauterine growth retardation (IUGR) incidences; GA; BW; delivery mode (vaginal delivery, VD; cesarean section, CS); gender (Male, M; Female, F); and Apgar scores at 1st and 5th min.

### 2.3. Standard Monitoring Procedures

Laboratory parameters (venous blood pH; partial venous pressure of carbon monoxide, pCO_2_; partial venous pressure of oxygen, pO_2_; base excess, BE; red blood cell count RBC; hemoglobin blood concentrations, Hb; hematocrit rate, Ht; glucose and ions) were recorded in all infants on admission to NICU.

### 2.4. BM Collection

Fresh Milk samples were collected into sterile, disposable, high-density polyethylene sealed bottles. Milk was collected with standard extraction methods by means of an electric breast pump (Medela Symphony, Baar, Switzerland). According to current guidelines, in order to collect full pumping samples, the extraction session was stopped 2 min after the outflow of the last drops of milk. From the total amount of milk of each mother, a sample of 5 mL of milk was collected and then stored at −80 °C until analysis.

### 2.5. P-SEP Measurements

PSEP milk levels were measured quantitatively (pg/mL) in 5 mL of BM. The detection was performed using a chemiluminescent enzyme immunoassay with the point-of-care automated analyzer PATHFAST-TM (Gepa Diagnostics, Milan, Italy) according to the manufacturer’s instructions. The assay detection limit was 200 pg/mL, the coefficient of variability intra-assay was ≤5.0%, and the coefficient of variability inter-assay was ≤10%.

The PATHFAST-TM analyzer was chosen for its high sensitivity and specificity for PSEP detection. Each 5 mL milk sample was determined to be optimal for ensuring consistent and reliable results based on preliminary trials.

### 2.6. Statistical Analysis

The demographic characteristics of maternal and neonatal outcomes were reported as mean ± SD. PSEP concentrations were expressed as median and interquartile ranges. Statistical analysis was performed by using a two-tailed paired t-test and a Mann–Whitney two-sided U-test when data did not follow a Gaussian distribution. A comparison between the groups was performed by using an ANOVA one-way test. Linear regression analysis was performed for correlations between PSEP levels in different milks and perinatal outcomes. Statistical analysis was performed using Sigma Stat 3.5 (GmBH, Mannheim, Germany). A *p* < 0.05 was considered significant.

## 3. Results

Perinatal characteristics and the main neonatal outcomes are reported in Table 1.

All enrolled mothers were Caucasian. Higher (*p* < 0.05, for all) incidences of GDM (6%), PIH (32%), and IUGR (18%) occurred in the PT group. As expected, GA and BW were lower (*p* < 0.05, for both) in the PT than in the T group. Moreover, the incidences of CS and VD (*p* < 0.05, for both) were higher and lower in the PT group, respectively. No differences (*p* > 0.05, for all) in maternal age, Apgar score at 1′–5′ min, or gender were observed between the studied groups.

Laboratory parameters recorded at admission to our NICUs are reported in Table 2. No significant differences (*p* > 0.05, for all) were observed between groups for blood, pH, pCO_2_, pO_2_, BE, RBC, Hb, Ht, blood glucose, or ion levels.

All infants admitted into the study were discharged in good clinical condition and no overt damage to any organs was detectable.

### 3.1. PSEP BM Levels in Total Population

**Figure 2:**
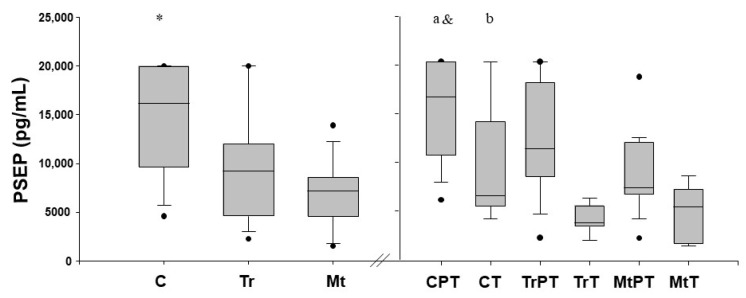
Presepsin (PSEP) milk levels (pg/mL) measured in colostrum (C), transition (Tr) and mature (Mt) milk fluids in the preterm (PT) and term (T) groups. Values are expressed in median and interquartile ranges. *p* < 0.05; * PSEP C vs. Tr vs. Mt; a PT PSEP C vs. Tr vs. Mt; b T PSEP C vs. Tr; & PT PSEP C, Tr, Mt vs. T PSEP C, Tr, Mt.

PSEP was detectable in all BM samples collected. When milk samples were corrected for the grade of maturation in the whole study population, the level of PSEP in C was higher (*p* < 0.001, for both) than in the Tr and Mt fluids. No significant differences (*p* > 0.05) were observed in PSEP between the Tr and Mt fluids (Figure 2).

### 3.2. PSEP BM Levels in PT and T Infants

The PSEP BM levels in the PT and T infants are reported in Figure 2. In the PT infants, the PSEP levels in the C fluid were significantly higher (*p* < 0.001) than those in the Tr and Mt, whilst no differences (*p* > 0.05) were detectable between the Tr and Mt fluids.

In T infants, the PSEP levels in the C fluid were significantly higher (*p* < 0.05) than those in Tr, whilst no differences (*p* > 0.05, for both) were detectable between C and Mt or between Tr and Mt.

Higher (*p* < 0.001) PSEP levels were observed in the PT compared to the T infants when comparing the total amount of the protein. Notably, when we compared PSEP levels between PT and T groups after correction for BM degree of maturation we found that PT infants had significantly higher (*p* < 0.001, for all) PSEP levels in C, Tr, and Mt fluids than their equivalent BM fluids in T infants (Figure 2).

### 3.3. PSEP BM and Delivery Mode

In Table 2, the PSEP levels when corrected for the delivery mode in the whole study population and for the BM sub-types are reported.

The PSEP levels in the VD PT and T infants were lower (*p* < 0.01) than in those born by CS. Conversely, higher (*p* < 0.001, for both) PSEP levels were found in PT infants delivered both by VD and CS than in T ones. No significant differences (*p* > 0.05, for all) in BM PSEP sub-types were observed among PT infants regardless of delivery mode. The same pattern (*p* > 0.05, for all) was observed in T infants. In PT VD infants, higher C PSEP than Mt was found, whilst no significant differences (*p* > 0.05, for both) between C and Tr or Tr and Mt were observed. In PT CS infants, the PSEP levels in the C fluid were significantly higher (*p* < 0.05, for both) than those in the Tr and Mt fluids, and the level of PSEP in Tr was higher (*p* > 0.05) than that in the Mt fluid.

In T VD infants, the PSEP levels were higher (*p* < 0.05) in C than Tr, whilst no differences were observed between C vs. Mt or Tr vs Mt.

After correction for CS, higher C PSEP and Tr (*p* < 0.05) than Mt levels were found (*p*< 0.05, for both). When we compared the levels of PSEP between the PT and T groups after correction for delivery mode and BM degree of maturation we found (i) higher (*p* < 0.05, for both) C and Tr PT VD PSEP levels than T ones, whilst no differences (*p* > 0.05) were detectable between Mt fluids, (ii) and higher (*p* < 0.05, for all) C, Tr and Mt PSEP levels in PT CS infants than T ones.

### 3.4. PSEP BM and Gender

Milk samples were corrected for gender in the whole study population and for the BM sub-types (Table 2). No significant gender differences (*p* > 0.05, for both) were observed in the PT and T total populations. Indeed, PSEP levels in the M and F PT sub-groups were higher (*p* < 0.001, for both) than M and F T male sub-groups.

In the M and F PT subgroups, we found higher (*p* < 0.05, for both) C PSEP levels than in the Tr and Mt fluids. No differences (*p* > 0.05) were found in PSEP between the Tr and Mt fluids.

In the M and F T subgroups, no differences (*p* > 0.05, for all) in PSEP levels among the C, Tr, and Mt fluids were found.

When we compared the PT and T groups after correcting for gender, we found higher (*p* < 0.05, for all) C, Tr, and MT PSEP levels in the M PT sub-groups than those of M T. Indeed, higher (*p* < 0.05, for all) C and Tr PSEP levels were observed in the F PT sub-group than the F T sub-group and no differences in Mt fluids between the sub-groups were shown. Finally, higher (*p* < 0.05) M PT Mt PSEP than F PT Mt PSEP was observed.

## 4. Discussion

The mother’s own milk is widely accepted as a unique fluid, containing biological factors involved in the regulation of optimal newborn development and growth (including hormones, growth factors, and cytokines), through which biochemical communication between mother and child is established [1,2,3,24]. This especially holds true for BM’s antimicrobial properties that protect particularly preterm infants from early and late infections and from major post-natal complications such as neonatal necrotizing enterocolitis [17]. The issue is of significance bearing in mind the high mortality and morbidity rates in that delicate population.

In the present study, we found that a promising biomarker for sepsis, namely PSEP, is more highly concentrated in the BM of mothers delivering a PT rather than a T infant. Notably, BM and its sub-types of maturation (colostrum, transition, mature) were delivery mode and gender dependent.

The presence of PSEP, or sCD14, and its sub-types at high levels in BM matches, in part, previous observations [28,29]. Discrepancies lie in the study population’s characteristics such as ethnicity, type of disease (bacterial and fungal sepsis, HIV), monitoring time-points (1 vs 4 months), and measurement technique (ELISA, CLEIA, cytometric bead array) [30,31]. Notably, none of the mothers were complicated by any allergy and/or excess maternal weight, both of which are known to affect PSEP release in the BM [32,33].

### 4.1. BM PSEP

The high PSEP levels detected in BM warrant further consideration. In particular, BM PSEP levels (i) were 10–20-fold higher than others detected in different biological fluids (i.e., blood, urine, saliva) from both of healthy PT and T infants and from those complicated by early/late onset sepsis [18]. This finding is in agreement with data showing that the levels of a series of cytokines involved in immune defenses, and in brain development [17], (ii) in both PT and T groups were higher than those previously reported. The explanation for this lies in the different measurement techniques and different endpoints used [17,29], and (iii) they were significantly increased in PT than T infants both considering total BM PSEP or its sub-type levels. This finding is noteworthy and offers additional support to the notion of the release of the marker for inflammation in placenta and systemic circulation during labor, particularly in PT cases known to be mainly triggered by a perinatal infection [34]. Bearing in mind that, in the present series, we excluded all the cases in whom perinatal sepsis had occurred, it is reasonable to argue for PSEP activation through a microbial-independent pathway [35].

### 4.2. BM PSEP Degree of Maturation

The finding that the PSEP levels increase according to the degree of BM maturation deserves further consideration. In particular, we found that the C PSEP levels in PT infants were higher than those measured at different stages of maturation (Tr, Mt), whilst in T infants a similar pattern was observed except there were no differences between the C and Mt fluids. These findings are in agreement with previous observations aimed at measuring a series of cytokines in BM and its sub-types [17,35]. Among a series of explanations, to date still under debate, the main ones, after having excluded environmental factors, regard functional/biochemical issues. In particular, (i) considering the significant differences in PSEP between the two main districts of concentration such as systemic circulation (low) and BM (high) the high C levels support the hypothesis of a mammary gland epithelium site of PSEP production [35], (ii) BM can achieve a PSEP serum extra-aliquot through a paracellular pathway [35], and (iii) PSEP over-production by mammary glands as a compensatory mechanism to adjust immunity of PT infants [29]. Altogether, the possibility that preterm birth, in the absence of any early laboratory signs of infection, could somewhat be triggered by a cascade of inflammatory events leading to the release of PSEP into systemic maternal circulation is, therefore, reasonable. Further studies aimed at elucidating the aforementioned issues are, therefore, justified.

### 4.3. BM PSEP and Delivery Mode

In the present study, we also found that BM PSEP levels both in PT and T infants were delivery mode dependent. In particular, the highest PSEP levels were observed (i) in PT infants compared to T ones regardless of delivery mode, and (ii) in C VD and CS PT sub-types both when compared to Tr and Mt PT and T BM fluids. Discrepancies with the literature data are due to the different PSEP measurement techniques, the study population (extremely PT vs. moderate and late PT infants), and monitoring time-points used [35]. The pattern of increased BM PSEP levels in PT born by VD and CS than T ones warrants further consideration. On the one hand, labor is associated with an increased immune response activation [36], whereas the increase in the immune factor in PT mother’s milk after labor could be a compensatory mechanism aiming to protect the immature infant [29].

### 4.4. PSEP BM and Gender

In the present series, we found that BM PSEP levels are gender dependent with higher Mt protein’ levels in PT males than females. To the best of our knowledge, the finding constitutes the first observation in this setting offering additional support to sex-related differences in the perinatal period [37]. Indeed, the higher impact of prematurity on male compared to female infants has also been demonstrated, as well as diversity in whole organ development [29]. The possibility that the higher Mt PSEP levels detected in PT male infants could be related to a pathophysiological cascade of events involving preterm labor, inflammation, and mammary gland PSEP over-release is, therefore, consistent. Thus, differences in the immune system will be included in a broader study aimed at investigating gender-related prenatal whole organ development.

### 4.5. Study Limitation

Lastly, the present study had a number of limitations such as (i) the small sample size, (ii) the lack of potential effects of maternal diseases on BM PSEP levels, and (iii) the potential bias due to maternal dietary regimen and pro-biotic supplementation on BM PSEP levels.

## 5. Conclusions

In conclusion, the present results showing the presence at high levels of PSEP in BM and its sub-types point to the protein’s potential antimicrobial role in the perinatal protection/development of the maternal–newborn dyad. Notably, the detection of high PSEP levels in PT C suggests its potential for early diagnostic use in neonatal care settings. Future studies should investigate the longitudinal effects of PSEP levels on infant health outcomes and the potential for integrating PSEP monitoring into routine neonatal screenings.

## Figures and Tables

**Table 1:** Characteristics of milk donors and perinatal parameters recorded in preterm (PT) and term (T) infants admitted into the study.

Parameters	PT (n = 22)	T (n = 22)	*p*
*Perinatal characteristics*			
Maternal age (yrs)	34 ± 5	33.9 ± 4	NS
GDM (n/tot)	5/22	1/22	<0.05
PIH (n/tot)	7/22	0/22	<0.05
IUGR	4/22	1/22	<0.05
*Neonatal characteristics*			
GA (wks)	33 ± 3.8	39 ± 4	<0.05
BW (g)	1950 ± 650	3100 ± 510	<0.05
CS (n/tot)	13/22	9/22	<0.05
VD (n/tot)	9/22	13/22	<0.05
Gender (M/F)	10/12	9/13	NS
Apgar 1′	8 ± 1	9 ± 0.9	NS
Apgar 5′	9 ± 1	9 ± 1	NS
*Monitoring parameters*			
pH > 7.20 (n/total)	22/22	22/22	NS
pCO_2_ (mmHg)	42 ± 3	41.6 ± 2.8	NS
pO_2_ (mmHg)	42.9 ± 1.2	43.1± 1.1	NS
BE	−1.1 ± 0.6	−1 ± 0.7	NS
RBC count (10^12^/L)	4.4 ± 0.3	4.3 ± 0.2	NS
Hb (g/L)	14.5 ± 3	13.8 ± 2.9	NS
Ht (%)	41 ± 0.07	41.5 ± 0.08	NS
Plasma glucose (mmol/L)	5.1 ± 0.2	5.2 ± 0.4	NS
Na^+^ (mmol/L)	138.5 ± 0.9	139 ± 1	NS
Ca^++^ (mmol/L)	1.12 ± 0.02	1.13 ± 0.03	NS
K^+^ (mmol/L)	3.8 ± 0.25	3.9 ± 0.2	NS

Abbreviations: PT, preterm; T, term; yrs, years; GDM, gestational diabetes mellitus; n, number; tot, total; PIH, pregnancy induced hypertension; IUGR, intrauterine growth retardation; GA, gestational age; wks, weeks; BW, birthweight; g, grams; CS, cesarean section; VD vaginal delivery; M, male; F, female; pCO_2_, partial carbon dioxide venous pressure; pO_2_, partial oxygen venous pressure; BE, base excess; RBC, red blood cell count; Hb, hemoglobin blood concentrations; Ht, hematocrit rate; Na+, sodium; Ca++, calcium; K+, potassium.

**Table 2:** Presepsin levels in preterm (PT) and term (T) infants corrected for delivery mode and gender. Values are expressed in median and interquartile ranges. *p* < 0.05: ^a^ PSEP C vs. Tr and Mt; ^b^ PSEP C vs. Tr; ^c^ PSEP Tr vs. Mt; ^d^ PSEP C vs. Mt; * PT C, Tr and Mt vs. TC, Tr and Mt; ** PT C and Tr vs. T C and Tr; ^&^ M Mt vs. F Mt.

	PT (n = 22)			T (n = 22)	
Presepsin (pg(mL)	Median	25°	75°	Median	25°
*Delivery Mode*					
C VD	15.906 ^d^**	11.326	19.789	5.843 ^b^*	4.545
Tr VD	9.185	2.356	16.014	3.836	2.741
Mt VD	6.458	5.501	7.502	6.334	2.125
C CS	16.718 ^a^	10.976	19.934	5.609 ^cd^	4.260
Tr CS	11.303 ^c^	9.228	19841	5.032	3.932
Mt CS	7.392	6.804	11938	1.738	1.637
*Gender*					
C M	16.447 ^a^*	11.314	19.471	6.596	5.051
Tr M	11.303	8.668	19.841	3.932	3.862
Mt M	9.800 ^&^	7.155	12.134	4.532	2.879
C F	16.888 ^a^**	10.638	19.879	5.609	3.877
Tr F	10.458	6.502	11.766	3.836	2.127
Mt F	6.804	6.555	7.391	6.334	1.681

Abbreviations: PT, preterm; T, term; C, colostrum; Tr, transition; Mt, mature; VD, vaginal delivery; CS, cesarean section; M, male; F, female.

## Data Availability

The data presented in this study are available on request from the corresponding author due to hospital privacy statement.
